# 517. Maternal RSV Vaccination and Infant Nirsevimab Coverage among Infants Born in the 2023-2024 Respiratory Virus Season in a Large Integrated Healthcare System

**DOI:** 10.1093/ofid/ofae631.169

**Published:** 2025-01-29

**Authors:** Karen B Jacobson, Andrew J Watson, Maqdooda Merchant, Bruce Fireman, Ousseny Zerbo, Nicola P Klein

**Affiliations:** Kaiser Permanente Vaccine Study Center, Oakland, California; Kaiser Permanente Vaccine Study Center, Oakland, California; Kaiser Permanente Vaccine Study Center, Oakland, California; Division of Research Kaiser Permanente Vaccine Study Center, Oakland, California; Division of Research Kaiser Permanente Vaccine Study Center, Oakland, California; Division of Research Kaiser Permanente Vaccine Study Center, Oakland, California

## Abstract

**Background:**

RSV is the leading cause of infant hospitalization in the United States. In 2023, the FDA approved two different measures to prevent respiratory syncytial virus (RSV) in healthy infants: a vaccine (RSVpreF) given to pregnant women at 32-36 weeks gestation, and a monoclonal antibody (nirsevimab) given to infants age < 8 months. Despite nationwide nirsevimab shortages, both it and RSVpreF vaccine were widely available to members of Kaiser Permanente Northern California (KPNC). We assessed coverage with either RSV preventative measure among infants born during the 2023-2024 respiratory virus season.

Percentage of Infants Protected against RSV by Birth Month within Kaiser Permanente Northern California, October 2023 – March 2024

**Figure:**
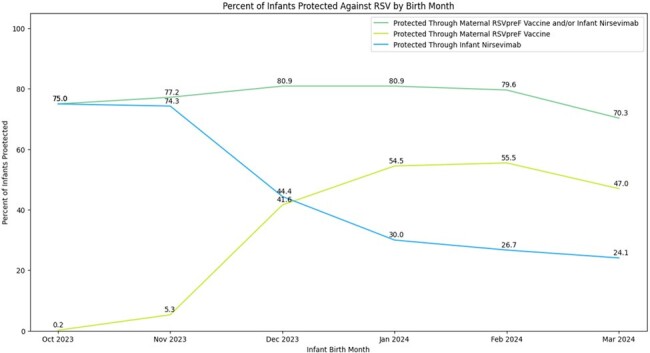

Among infants born in October 2023, 0.2% were protected through maternal RSVpreF vaccine during pregnancy, and 75.0% were protected through nirsevimab received after birth. Among infants born in January-March 2024, >50% were protected through maternal RSVpreF, while <30% were protected through nirsevimab. Overall, however, 77% of infants born in the 2023-2024 respiratory virus season received protection from RSV through either maternal RSVpreF vaccination in pregnancy and/or nirsevimab administration in infancy.

**Methods:**

Using KPNC’s electronic health records, we describe maternal RSVpreF vaccination and infant nirsevimab administration among infants born to mothers age 15-49 years at KPNC facilities between October 17, 2023 and March 31, 2024. In KPNC, Nirsevimab infusions began October 17, 2023 and maternal RSVpreF vaccinations began October 25, 2023. We compared infants protected against RSV, through maternal RSVpreF and/or infant nirsevimab, to unprotected infants.

**Results:**

Of 17,251 infants included, 13,365 (77.5%) received at least one method of RSV protection as of May 4, 2024. 6,314 (36.6%) infants were born to mothers vaccinated against RSV during pregnancy at median gestational age 239 (IQR 228, 252) days. Median days from RSVpreF to delivery was 33 (23, 45). 7,511 (43.5%) infants received nirsevimab at median age 4 (IQR 2, 16) days. Of 460 (2.7%) infants protected by both maternal RSVpreF and nirsevimab, 143 (31.1%) were born preterm and 111 (24.1%) spent time in NICU. Compared to unprotected infants, RSV protected infants were born to older mothers (age 31.4 [SD 5.1] vs 30.6 [SD 5.2] years, p< 0.0001). Overall RSV protection was highest in infants with Asian mothers (86.7%) and lowest in those with Black mothers (70.2%).

**Conclusion:**

In an integrated healthcare system with ample supply, nearly 80% of infants born during the respiratory season received RSV protection through either maternal RSVpreF vaccination or infant nirsevimab. Infants of mothers who were older or Asian race were more likely to be protected against RSV. Further research should examine how to reduce racial disparities in RSV protection coverage.

**Disclosures:**

**Nicola P. Klein, MD, PhD**, GlaxoSmithKline: Grant/Research Support|Merck: Grant/Research Support|Pfizer: Grant/Research Support|Sanofi Pasteur: Grant/Research Support|Seqirus: Grant/Research Support

